# Testicular decompression and tunica vaginalis flap in human acute testicular torsion: modified step-by-step technique description and preliminary outcomes

**DOI:** 10.31744/einstein_journal/2023AO0220

**Published:** 2023-08-03

**Authors:** Alexandre Kyoshi Hidaka, Felipe Placo Araújo Glina, Renan Murata Hayashi, Khalil Smaidi, Willy Baccaglini, Cristiano Linck Pazetto, Fabio José Nascimento, Sidney Glina

**Affiliations:** 1 Centro Universitário FMABC Santo André SP Brazil Centro Universitário FMABC, Santo André, SP, Brazil.

**Keywords:** Spermatic cord torsion, Compartment syndromes, Testis, Testicular diseases, Pressure, Urologic surgical procedures, male

## Abstract

**Objective:**

To report the effects of a tunica vagina flap on testicular compartment syndrome.

**Methods:**

This single-arm clinical trial was conducted from September 2020 to October 2021. Consecutive patients with suspected testicular torsion within 24 hours of pain onset were included. Patients with past testicular torsion, bilateral torsion, or previous atrophy were excluded. The tunica vaginalis was opened, and the intratesticular pressure was measured before testicular retrieval from the scrotum and detorsion (P1), after detorsion (P2), and after transverse incision (P3). A tunica vaginalis flap was performed and a new intratesticular pressure was recorded (P4). The contralateral testicular pressure was recorded before fixation (Pc). The minimum follow-up period was 6 months.

**Results:**

Fifteen patients were recruited from September 2020 to October 2021. Nine patients completed the follow-up. The median age (range) was 15 years (9-19). The mean P1, P2, P3, P4, and Pc (range) were 43, 60, 23, 20, and 14mmHg, respectively. The atrophy rate was 66.3% and the viability was 88.9%. No major complications were observed.

**Conclusion:**

The modified tunica vaginalis flap in acute testicular torsion decreased intratesticular pressure. Furthermore, normal testicular pressure can improve testicular preservation. It can also decrease testicular pressure to normal levels and preserve the testicular parenchyma.

## Highlights

Testicular torsion causes orchiectomy in more than 80% of the cases after 24 hours of pain onset.

Testicular detorsion only (P1-P2) showed a small difference in intratesticular pressure (median 4mmHg).

Testicular incision (P2-P3) only demonstrated a major pressure decrease (median, 45mmHg).

The final pressure after TVR was similar to the non-twisted testicle pressure (median, 1mmHg).

Testicular salvage (88.9%) and atrophy (66.3%) rates were slightly higher than the previous literature reports, perhaps the initial learning curve of this novel technique.

**Figure:**
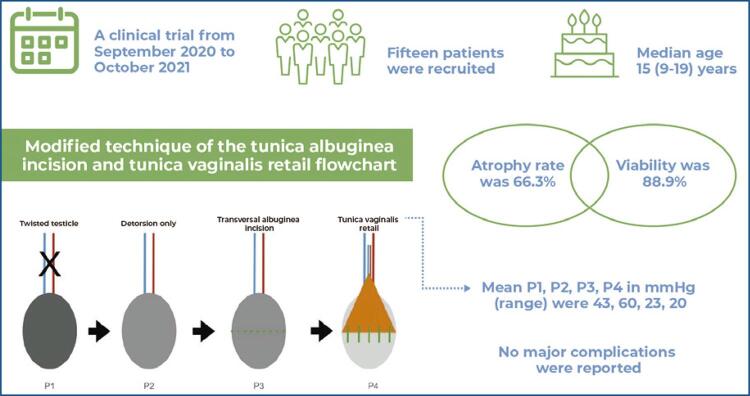

## INTRODUCTION

Testicular torsion is an emergency situation, and the time to treatment affects the testicular salvage rate in the first 24 hours after pain onset. It occurs in one in every 4,000 men within 25 years.^(1)^ Time and degree of torsion are the main predictors of testicular death. Scrotal exploration is recommended within 4-6 hours of pain onset to maximize the testicular salvage rate. After 24 hours, the testicular death rate is >90%.^(2)^

Compartment syndromes associated with testicular torsion have been debated over the past few decades. Its exact mechanism of action remains unclear. However, torsion causes an abrupt reduction of the venous blood efflux from the testis leading to intratesticular swelling and damage to the parenchyma and micro-circulation.^(3)^

Regarding oxidative stress, leucocytes contribute to the production of reactive oxygen species (ROS).^(4)^ These harmful substances have deleterious effects on the structure and function of cells.^(5)^ Under physiological conditions, antioxidative enzymes can modulate the synthesis of ROS to protect organs and cells against oxidative stress.^(6)^ In ischemic injury, an imbalance between the antioxidant system and produced ROS reduces the activity and content of antioxidative enzymes, thereby increasing the levels of ROS.^(7,8)^

During surgical exploration, the testis is considered unsalvable if the ischemic or necrotic aspects do not improve after detorsion and heating. In these cases, orchiectomy and contralateral orchiopexy are the standard treatments.

To validate the tunica vaginalis flap (TVF) technique in humans and prove compartment syndrome in testicular torsion, Kutikov et al. reported the TVF.^(9)^

## OBJECTIVE

To evaluate the safety, feasibility, and intratesticular pressure effect of the tunica vaginalis flap modification technique and to report our preliminary outcomes.

## METHODS

After counseling and consent, patients with suspected unilateral testicular torsion within 24 hours were included in this study. The underage patients’ parents or legal guardians were counseled and they agreed. The exclusion criteria were bilateral torsion, previous torsion, contralateral atrophy, and complete restoration of testicular morphology after detorsion.

This was a prospective, single-arm, unicentric clinical trial. The study was approved by the ethics committee of *Centro Universitário* FMABC (CAAE: 34354920.5.0000.0082; # 4.184.227).

### Intratesticular pressure measurement (IPM)

**Figure 1:**
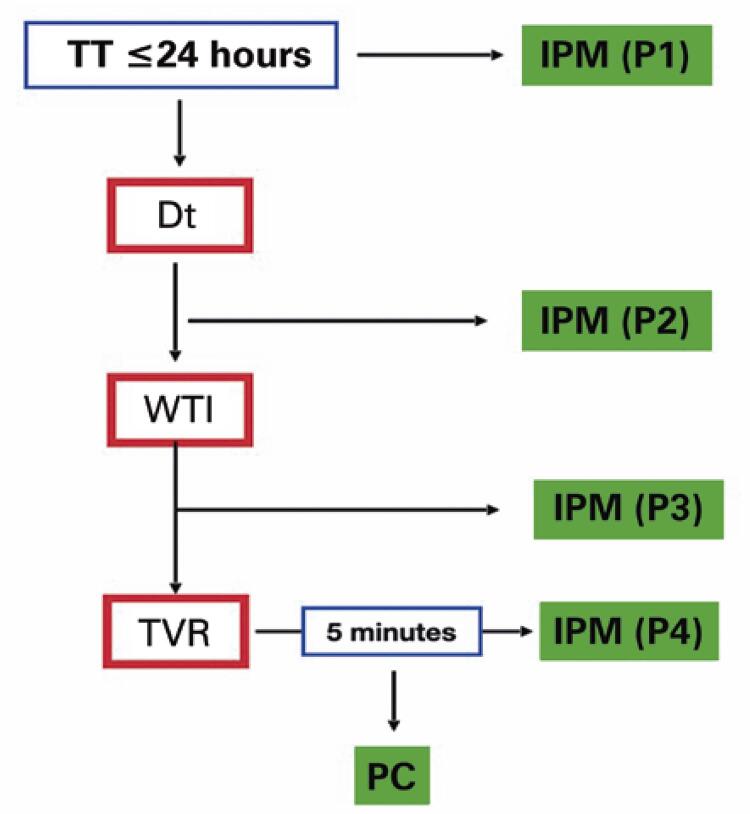
Intratesticular pressure measurement templates TT: testicular torsion; IPM: intratesticular pressure measurement; Dt: detorsion; WTI: wide transverse incision; TVF: tunica vaginalis flap; PC: contralateral testicular pressure.

All IPMs were performed concurrently under anesthesia. No longer than 10 seconds was required per measurement. An electronic vital sign monitor from the operating room was adapted to measure ITP since there is no authorized device for proper use in Brazil. The same invasive technique was used to measure arterial pressure. A venous punction 20-G catheter was used to puncture the testes after injection of 0.3mL of saline solution. Standard arterial pressure tubing was placed and connected to a puncture catheter. The mean pressure (mmHg) was recorded. Intratesticular pressure measurement were recorded at four moments: before and after detorsion, after albuginea incision, and after TVF. Non-twisted testicle was punctured at the same location as the fixation stitch (Figure 1).

### Surgical technique

**Figure 2:**
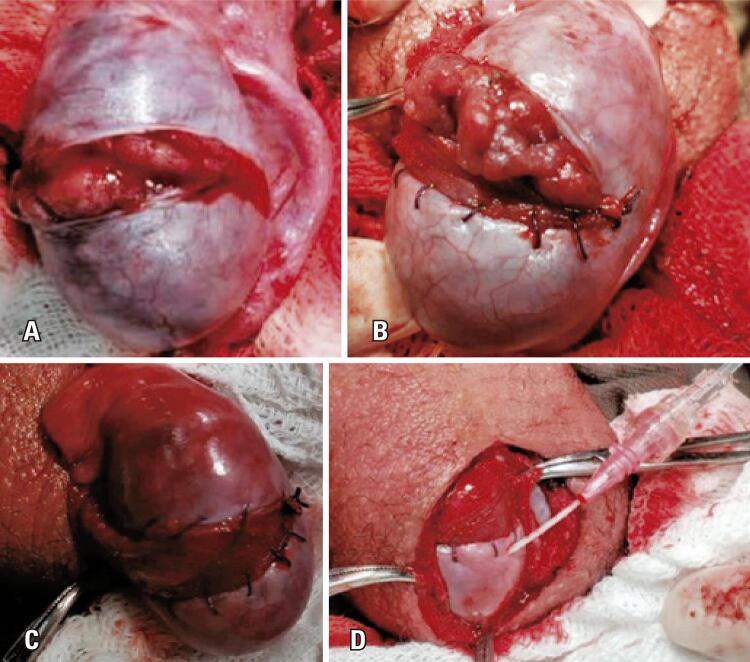
Surgical technique. A) Wide transverse incision; 2B and 2C) Continuous running suture with 4-0 polyglactin wire; 2D) Intratesticular pressure measurement after tunica vaginalis flap (P4)

A standard median scrotal incision was made. The tunica vaginalis was opened lengthwise, and the IPM was measured before testicular retrieval from the scrotum and detorsion (P1). Testis detorsion and heat were applied. A contralateral orchiopexy was performed. The second IPM was recorded (P2). Wide transverse incision (WTI) was performed. This step was a modification of the original description by Kutikov et al.^(9)^ (Figure 2A) to preserve the transversal vessels of the albuginea. Active arterial bleeding from the testicular parenchyma might have been evident at this point. The third IPM was recorded (P3). A TVF was created using a simple running suture with an absorbable 4-0 polyglactin wire (Figures 2B and 2C). After completion, the fourth IPM was performed (P4) (Figure 2D). The detorsed testis was fixed at three points with two stitches in the dark muscle (one distal and one lateral) and the TVF. The TVF also fixed the testes in the medial portion. The contralateral testicular pressure was recorded before orchidopexy (Pc). No drain was placed.

### Tunica vaginalis flap

**Figure 3:**
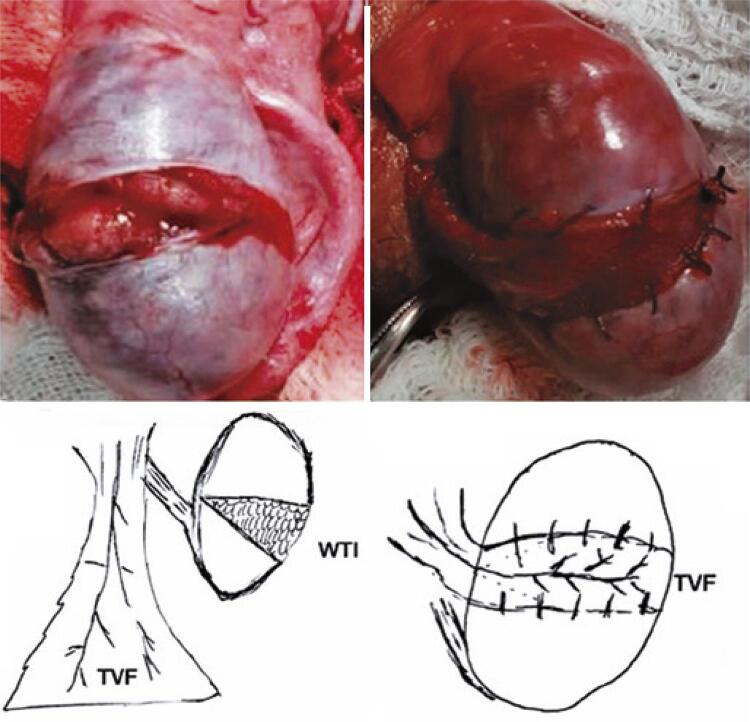
Tunica vaginalis flap confection and testis fixation with an absorbable wire continuous running suture WTI: wide transverse incision; TVF: tunica vaginalis flap.

To decrease the ITP and promote wide flap coverage, the vascular pedicle must be preserved from the vaginal flap (Figure 3). The rationale behind the coverage was to prevent parenchymal exposure to reduce oxidation and ROS levels.

### Post surgery care

All patients were discharged with a standard orientation. Scrotal suspension was recommended for 14 days. First-generation cephalosporins were prescribed for 7 days postoperatively. If any sign of wound infection was observed, the medication was postponed or changed to quinolone for additional 7 days.

### Follow up

Intrahospital, 7 days post hospital discharge, and 2 and 6 months postoperative complications were recorded during routine medical presentation. Doppler ultrasonography and flowmetry were performed 2 months postoperatively. Atrophy was measured using a physical examination (orchidometer) and an ultrasound report.

### Outcomes

The primary outcome was the effect of TVF on testicular pressure.

The secondary outcomes were the safety, feasibility, and reproducibility of the modified TVF technique. To define testicular atrophy and salvage rates, atrophy was considered if the affected testicle contained <50% of the volume of the non-twisted testicle. Following previous reports,^(9-11)^ this outcome was dichotomized into any atrophy or none. Viability was defined as a normal testicular blood flow on Doppler ultrasonography.

## RESULTS

Fifteen consecutive patients were recruited from September 2020 to October 2021. Three patients were excluded from the surgical procedure because of complete morphological recovery of the testicle, and bilateral orchidopexy was performed. Two patients who had no testicular torsion were excluded from the study. Eleven patients were eligible for the TVF therapy. Nine patients completed the 6 months follow up and were considered for statistical analysis. The median age (range) was 15 years (9-19). The right side was affected in 70% of the patients. The mean degree of torsion (range) was 180 (180-360).

The mean P1, P2, P3, P4, and Pc were 43, 60, 23, 20, and 14mmHg (range), respectively. The surgical pressure step differences are presented in table 1. The atrophy rate was 66.6%. The testicular salvage rate was 88.9%. Ultrasound and physical examination volume measurements (mL) are presented in table 2.

**Table 1:** Intratesticular pressure measurement (mmHg)

Patients	P1	P2	P3	P4	PC	P1-P2	P2-P3	P1-P4	P2-P4	P3-P4	PC - P4
1	MD	60	33	20	MD	MD	27	MD	40	13	MD
2	60	40	40	45	MD	20	0	15	-5	-5	MD
3	40	30	20	13	22	10	10	27	17	7	-9
4	25	24	23	21	9	1	1	4	3	2	12
5	41	121	76	51	67	-80	45	-10	70	25	-16
6	60	53	7	8	4	7	46	52	45	-1	4
7	MD	63	13	14	13	MD	50	MD	49	-1	1
8	45	140	15	14	14	-95	125	31	126	1	0
9	MD	82	36	35	32	MD	46	MD	47	1	3
Median	43	60	23	20	14	4	45	21	45	1	1

P1: before detorsion; P2: after heating and detorsion; P3: after transverse albuginea incision; P4: after tunica vaginalis retail; PC; contralateral (non-twisted); MD: missing data.

**Table 2:** Atrophy rate

Patients	T4 (h)	Degree (^o^)	US flow	Torsion (mL)	Normal (mL)	N-T	Atrophy rate (%)
1	7	180	N	18	13	-5	138.5
2	14	180	N	4.0	22.6	18.6	17.7
3	21	180	N	8.9	4.1	-4.8	217.1
4	6	180	D	1.6	3.5	1.9	45.7
5	8	180	N	1.8	10.9	9.1	16.5
6	6	360	N	3.6	11.6	8.0	31.0
7	6	180	N	4.4	2.3	-2.1	191.3
8	9	360	N	3.6	10.0	6.4	36.0
9	2	180	N	3.8	8.7	4.9	43.7
Median	7	180	-	3.8	10.0	4.9	43.7

T4: time from pain onset to surgical intervention; degree, torsion degree; US flow: Doppler flowmetry; N-T: non-twisted testicle.

No major complications were observed. No testicular abscesses, hematomas, or wound infections were observed. Only one case of wound dehiscence that resolved after 2 months was reported.

## DISCUSSION

Prolonged or even short ischemia causes tissue damage and necrosis, which can produce high levels of oxidative stress in the testes, especially during the reperfusion period. Testicular torsion/detorsion increases lipid peroxidation and decreases antioxidant enzyme levels. Increased levels of peroxidase enzyme injury after testicular reperfusion can induce molecular damage to the DNA.^(3)^

To validate the TVF technique in humans and prove the compartment syndrome in testicular torsion, Kutikov et al. reported TVF in three cases. All treated 4-7 hours after the onset of pain. Testes without aspect improvement after detorsion and heating and those with active parenchymal bleeding after a wide anterior longitudinal albuginea incision were candidates for TVF. Intratesticular pressure was measured in only one patient and decreased from 34mmHg before detorsion to 5mmhg after TVF correction. Atrophy was reported in one case and Doppler flowmetry was positive in two cases.^(9)^

The initial technique, described in 2018^(8)^ advocates a wide longitudinal incision on the anti-epididymal side. However, considering that the albuginea vessels run transversally, our technique was modified to make a wide transverse incision in the avascular equatorial area of the testis to preserve the albuginea vessels.

In this series, all cases experienced a decrease in ITP before and after TVF. To investigate the impact of the detorsion maneuver only (P1-P2), we measured the pressure before (P1) and after detorsion and heating (P2). Most patients exhibited a slight decrease in ITP, with a median value of 4mmHg. Interestingly, detorsion did not lower the ITP in many cases. A major decrease occurred after the albuginea incision (P2-P3) with a median value of 45mmHg. The most important outcome was approximately the same pressure after TVF and the contralateral testicle, with a median range of 1mmHg. Based on our results, we concluded that TVF may achieve normal testicular pressure, which may improve testicular reperfusion. The puncture of non-twisted testicles appears to be safe. Once the Pc puncture point was planned to be in the same place as the fixation stitch, no minor or major complications regarding twisted or non-twisted organs were recorded in this study.

In 2012, Figueroa et al. reported a retrospective analysis, where the TVF was compared between orchidopexy and orchiectomy groups. They applied TVF in selected cases within 96 hours of pain onset. The ITP was not assessed in this study. The testicular salvage rate in the TVF group was 54.6% (p=0.19), being successful within a median of 31.2 hours of pain onset. This report revealed a longer treatment time with preservation of nearly 50% of the testicular candidates for orchiectomy.^(10)^ In 2018, Chu et al.^(11)^ performed a retrospective analysis; the results were similar to those reported by Figueroa et al.^(10)^ The study showed testicular salvage and atrophy rates <24 hours and >24 hours from pain onset of 95% TVF and 67% *versus* 67% TVF and 83%, respectively.^(11)^ Both authors reported no major complications.

However, a recent meta-analysis of orchidopexy techniques demonstrated an ipsilateral atrophy rate in only three studies, ranging from 9.1% to 47.5%.^(12)^ They included the retail technique reported by Figueroa et al.^(10)^ which showed the highest atrophy rate. Despite the lack of well-designed studies with longer and proper follow-up periods, there is an apparent overall lower atrophy rate. There is no strong evidence for testicular viability in the literature.

A clinical trial using TVF in a rat model demonstrated a reduction in ROS levels. Oxidative stress was assessed using malondialdehyde levels in the 1 *versus* 5 *versus* 9 hours groups. The best recovery was observed in the 9 hours group.^(13)^

In this clinical trial, prospective evaluation revealed a high atrophy rate of 66.6%, with a similar testicular salvage rate of 88.9%. Despite documented decompression, this high rate may be associated with the initial learning curve and lack of initial standardization of pressure measurement and surgical technique. This pilot project standardized TVF in a university hospital.

### Limitations

Initially, our university hospital had only one experienced surgeon with expertise in this approach. The first cases were documented for educational purposes by all the department surgeons. This initial learning curve might have affected our outcomes negatively. Second, owing to the lack of approved medical tools to properly measure the ITP, we adapted a surgical monitor and an invasive pressure tool. Similar to the initial surgical learning curve, pressure measurements might have affected the initial pressure outcomes. Therefore, to minimize this bias, the differences between the pressures of each patient were used. It is not possible to define normal human testicular pressure using this measurement tool. Third, only a few patients were included in the statistical analysis. Regarding the public health system of our city and interhospital transportation needs, few patients reached a urologist within 24 hours of pain onset.

To address these initial difficulties, we aim to conduct a multicenter randomized controlled trial in the near future. Perhaps a prospective randomized trial comparing the flap group *versus* orchiopexy alone may elucidate the real benefit of this technique in orchiectomy candidates.

## CONCLUSION

The modified tunica vaginalis flap seems to decrease intratesticular pressure in acute testicular torsion. Normal testicular pressure can be used to improve testicular preservation. It can decrease testicular pressure to normal levels and preserve the testicular parenchyma. Further studies are required to validate these results.
